# Inhalant use in adolescents in northern Russia

**DOI:** 10.1007/s00127-018-1524-z

**Published:** 2018-05-02

**Authors:** Roman Koposov, Andrew Stickley, Vladislav Ruchkin

**Affiliations:** 1 Regional Center for Child and Youth Mental Health and Child Welfare, UiT The Arctic University of Norway, Tromsö, Norway; 2 The Stockholm Center for Health and Social Change (SCOHOST), Södertörn University, Huddinge, Sweden; 3 Child and Adolescent Psychiatry Unit, Department of Neuroscience, Uppsala University, 751 85 Uppsala, Sweden; 4 Child Study Center, Yale University Medical School, New Haven, CT USA; 5 Säter Forensic Psychiatric Clinic, Säter, Sweden

**Keywords:** Inhalant use, Adolescents, Mental health

## Abstract

**Purpose:**

To determine the prevalence of inhalant use in Russian adolescents and to investigate associated psychosocial problems from a gender perspective.

**Methods:**

Data on inhalant use and comorbid psychopathology were collected by means of self-reports from 2892 (42.4% boys) sixth to tenth grade students in public schools in Arkhangelsk, Russia. Multivariate analysis of covariance was used to assess differences in the levels of internalizing and externalizing problems in boys and girls, who were non-users and users of inhalants.

**Results:**

The prevalence of inhalant use was 6.1% among boys and 3.4% among girls. Compared with non-users, inhalant users scored significantly higher on internalizing and externalizing problems, functional impairment and lower on academic motivation, with psychopathology increasing with age. While there were no gender differences for internalizing problems, increased levels of externalizing problems in inhalant users were gender-specific (significantly higher in boys).

**Conclusions:**

Inhalant use is related to significantly higher levels of comorbid psychopathology in Russian adolescents. Comprehensive, evidence-based prevention and intervention policies are needed to address inhalant use and its harmful effects.

## Introduction

Substance use is common among adolescents [1, 2]. Among substances, inhalants (household and industrial chemicals) occupy a special place because they are relatively inexpensive and readily available. Inhalants are often among the first and most commonly used substances aside from alcohol and cigarettes [3]. Inhalant use often starts at an early age, in almost half of the cases, before 13 years of age, and in over 80% of users before age 15 [4]. Research suggests that inhalant use rates peak during the mid-teenage years, with the rate of use among 14 year olds (3.4%) being nearly twice that of 12 year olds (1.8%) [5].

Inhalants are also among the most dangerous types of drugs abused by children and adolescents, as they are highly addictive both physically and psychologically [6, 7] and there is a substantial risk of a permanent impact on mental and physical functioning [8]. Even experimental use of inhalants has been linked to adolescent risk-taking and disruptive behaviors [9, 10], whereas the chronic use may lead to more serious and long-lasting mental and somatic health problems [11, 12]. Studies of adolescent inhalant users have found that they are more likely than non-users to report poor family relations, disrupted living situations, academic problems [13, 14] and are at increased risk of multiple drug use [13]. Furthermore, inhalant use has been linked to higher rates of anxiety and depression [7], suicidal thoughts and attempts [15, 16], as well as to delinquency [17].

Despite extensive research on youth inhalant use in the United States [4, 18], very few large-scale studies have been conducted to date on this phenomenon in countries outside the US. In Europe, the reported rates of ever using inhalants for students aged 15–16 years old have varied widely from 3% in Bulgaria to 18% in Ireland [19]. Data from the European School Survey Project on Alcohol and Other Drugs (ESPAD) showed that in Russia the prevalence of inhalant use in the general adolescent population was 9% [20]. However, other reports suggest a higher figure, for example, statistics from the Russian Ministry of Education showed that 16% of adolescents and young adults used inhalants regularly (2–3 times a week) [21].

Considering the lack of large-scale epidemiological data and a high discrepancy in the reported figures, more research is needed to obtain a clearer picture of the situation regarding inhalant use and its effects in Russian adolescents. In addition, given the substantial gender differences in the prevalence of internalizing and externalizing problems in adolescents [22], it is possible that the use of inhalants may be associated with different types of problems in boys and girls. Hence, this study had two main aims, namely, (1) to assess the general prevalence of inhalant use in Russian adolescents, and (2) to investigate the psychosocial problems associated with inhalant use in Russian youths from a gender perspective.

## Methods

### Sample and procedures

Data were drawn from a survey undertaken as part of the Social and Health Assessment (SAHA) research project [23, 24]. The survey was conducted in the northwestern Russian city of Arkhangelsk. Data were collected from a representative sample of students (age 12–17) in the city’s public schools (i.e. 10% of students in each of the city’s four districts). The subjects were selected from schools that were randomly selected from a list of all the city’s schools, and from within classes randomly selected from within each school. All invited students completed the survey questionnaire anonymously in their classrooms in the presence of teachers during a normal school day. Written informed consent was obtained before the questionnaire was administered and children had the right to refuse to participate in the study. The recruited sample comprised 2892 students (42.4% boys) with a refusal rate of 3.6%. Ethical approval for the study was obtained from the Northern State Medical University in Arkhangelsk, Russia.

### Measures

Items from the SAHA survey questionnaire were used in the study. The survey included new scales that were developed specifically for the SAHA study and scales that had been used previously with similar populations [25, 26]. In the current study, the following measures were used.

#### Inhalant use

The students were asked to indicate how often they used huffing (glue, aerosols) to get high on a 5-point response scale (“Never”, “1–2 times a year”, “1–2 times a month”, “1–2 times a week”, and “Every day”). The respondents were then divided into users and non-users.

#### Internalizing problems

*Depressive symptoms* were assessed using an adaptation of the Center for Epidemiologic Studies Depression Scale (CES-D) [27] consisting of ten items (e.g. “I felt really down”). Respondents reported on the presence of depressive symptoms during the past month on a three-point scale [“Not true” (scored 0); “Somewhat true” (1); or “Certainly true” (2)]. The total score ranged from 0 to 20 with higher scores indicating increased depressive symptoms (Cronbach’s *α* for the scale = 0.80).

*Anxiety symptoms* were assessed with 12 items [23] describing worrisome, preoccupying thoughts or unpleasant feelings about oneself or external stimuli (e.g. “I worry about other people liking me”). Students reported on the presence of anxiety symptoms on a three-point scale [“Not true” (scored 0); “Somewhat true” (1); or “Certainly true” (2)]. The total score ranged from 0 to 24 with higher scores indicating increased anxiety symptoms (*α* = 0.87).

*Posttraumatic stress symptoms* were assessed with the Child Post-Traumatic Stress-Reaction Index (CPTS-RI; [28]), a self-report questionnaire that contains 20 items with a Likert-type response scale assessing the frequency of symptoms ranging from “Never” (0), to “Most of the time” (4). The CPTS-RI total score (ranging from 0 to 80) has been found to correspond highly with the clinical diagnosis of PTSD [29] (*α* = 0.85).

*Impact Supplement of the Strength and Difficulties Questionnaire* (SDQ) [30] is a brief screening questionnaire that asks whether the respondent (1) thinks that he/she has difficulties in emotions, concentration, behavior, or in getting along with other people (ranging on a 4-point scale from “No” to “Yes, severe difficulties”), and (2) if so, about the perceived degree of distress (“Do these difficulties upset or distress you?”); of social impairment (“Do these difficulties interfere with your everyday life?” in four areas: home life, friendships, classroom learning, and leisure activities); and of burden to others (“Do these difficulties make it harder for those around you?”). Responses for distress, social impairment, and burden to others ranged on a 4-point scale from “Not at all” (0) to “A great deal” (3). A total impact scale score was calculated by summing up the six items, covering perceived distress, social impairment in 4 different domains and burden to others. In cases in which the response was ‘No’ to the first impact question, the whole impact score was set to zero (*α* = 0.73).

#### Externalizing problems

*Alcohol use* was assessed with three items derived from the Monitoring the Future Scale [31] that quantified the use of three alcoholic beverages (beer, wine, and hard liquor) during the last 30 days (using a 4-point scale, ranging from “0” to “6 or more times”) (*α* = 0.88).

*Marijuana use* was assessed with a single question, asking the respondent to indicate the frequency of use during the last 30 days (using a 4-point scale, ranging from “0” to “6 or more times”).

*Problems related to alcohol use* [23]. This 11-item scale asks whether the respondent had problems in the past year related to drinking, such as getting into fights, having money problems, damaged friendships, etc. All items are answered on a 4-point scale ranging from “0” to “6 or more times” (*α* = 0.74).

*Problems related to substance use* [23]. This scale consists of five items, asking whether the respondent ever had problems related to the use of drugs, such as getting into an argument, feeling sick, getting arrested, etc. The items are rated on a 3-point scale (from “Never” to “Often”). The total score ranged from 0 to 10 with higher scores indicating increased problems (*α* = 0.72).

*Antisocial Behavior Scale* [32]. This includes three subscales assessing behavior problems of different severity. Respondents are asked to report on a 5-point scale how many times (ranging from “0” to “5 or more times”) they were involved in the following behaviors during the past year. (1) *Conduct Problems*. Includes six items describing mild behavior problems, such as lying to a teacher or a parent, staying out all night without permission, shoplifting, etc. The total score ranged from 0 to 24 with higher scores indicting greater problems (*α* = 0.78). (2) *Less Severe Delinquency*. This subscale consists of five items describing non-violent antisocial behavior, such as stealing a motorcycle/car, pick-pocketing etc. The total score ranged from 0 to 20 with higher scores indicating greater delinquency (*α* = 0.75). (3) *Severe Delinquency*. This consists of five items, pertaining to relatively serious aggressive and antisocial behaviors, i.e. starting a fistfight, being arrested by the police, etc. Students were asked to report on a 5-point scale how many times they had been involved in each type of antisocial behavior during the past year (from 0 = zero times to 4 = five or more times). The total score ranged from 0 to 20 with higher scores indicating higher levels of severe delinquency (*α* = 0.78).

*Academic Motivation Scale* [33, 34]. This included six items describing the perceived importance of academic achievement and academic motivation (e.g. “It is important to me to be considered a bright student by my teachers”). The items are rated on a 4-point scale, ranging from “Definitely not true” (1) to “Definitely true” (4). The total score ranged from 6 to 24 with higher scores indicating higher levels of academic motivation (*α* = 0.84).

*Affiliation with delinquent peers* For this 9-item scale developed by the SAHA Research Evaluation Team [23], respondents are asked how many of their close friends (from “None” to “Most or all”) are involved in different types of delinquent behavior such as dropping out of school, smoking cigarettes, drinking alcohol, etc. The summed score could range from 9 to 36 with higher scores indicating higher levels of affiliation with delinquent peers (*α* = 0.76).

### Statistical analysis

Multivariate analysis of covariance (MANCOVA) was used to assess differences in the levels of internalizing and externalizing conditions/behaviors in boy and girl users and non-users of inhalants. Hence, we used a 2 (inhalant use) by 2 (gender) design conducted for internalizing and externalizing problems separately to diminish the number of variables in the analysis and to avoid multiple comparisons. Because age influences children’s development and is associated with the outcome variables in this study, all analyses were conducted while controlling for age. The unique contribution of each of the two fixed factors (inhalant use and gender), the covariate (age), and the one interaction term (inhalant use × gender) was assessed through post hoc between-subject tests and unstandardized parameter estimates derived from the MANCOVA. Results are presented as means (*M*) and standard deviations (SD), and for individual outcomes, as partial eta squared (*η*^2^), a common metric of effect size that represents the unique amount of variance explained by each predictor variable.

## Results

The prevalence of inhalant use was 6.1% (*n* = 69) among boys and 3.5% (*n* = 55) among girls. Of these, 3.6% (*n* = 41) of boys and 2.4% (*n* = 39) of girls used inhalants irregularly, while 2.5% (*n* = 28) of boys and 1.0% (*n* = 16) of girls reported using inhalants several times a week. Given the relatively low prevalence rates, both the irregular and regular inhalant user groups were combined into a single users group, which was then compared to the non-users group.

Tables 1 and 2 present descriptive statistics [*M* (SD)] from the MANCOVA regarding differences in internalizing and externalizing problems, respectively, by boys’ and girls’ inhalant use. Compared with non-users, inhalant users of both genders scored significantly higher on depression, posttraumatic stress, functional impairment and lower on academic motivation. Inhalant users also had higher scores on alcohol and marijuana use, alcohol and substance use-related problems, conduct problems, delinquency and reported a higher affiliation with delinquent peers.

**Table 1:** Internalizing problems scores [*M* (SD)] by gender in relation to inhalant use

Variable	Non-users (*n* = 2595)		Users (*n* = 124)	
	Boys (*n* = 1070)	Girls (*n* = 1525)	Boys (*n* = 69)	Girls (*n* = 55)
Depression	4.95 (3.91)	6.60 (4.21)	7.50 (5.35)	7.75 (4.33)
Anxiety	12.22 (5.82)	14.11 (5.52)	13.22 (4.77)	13.19 (5.43)
Posttraumatic stress	17.42 (10.78)	21.33 (11.38)	25.70 (15.66)	24.60 (13.65)
Academic motivation	17.03 (3.10)	17.48 (2.93)	16.08 (2.69)	15.88 (3.40)
SDQ functional impairment	0.69 (1.43)	0.92 (1.62)	1.78 (2.51)	1.85 (2.39)

*M* mean, *SD* standard deviation

**Table 2:** Externalizing problems scores [*M* (SD)] by gender in relation to inhalant use

Variable	Non-users (*n* = 2499)		Users (*n* = 95)	
	Boys (*n* = 991)	Girls (*n* = 1508)	Boys (*n* = 47)	Girls (*n* = 48)
Alcohol use	4.37 (3.08)	4.35 (2.98)	6.85 (2.82)	6.35 (2.46)
Alcohol-related problems	1.22 (1.82)	0.93 (1.48)	4.11 (2.82)	2.67 (2.08)
Marijuana use	0.24 (0.84)	0.14 (0.53)	1.87 (2.15)	1.04 (1.80)
Substance use-related problems	0.12 (0.55)	0.06 (0.41)	2.49 (2.77)	1.60 (2.58)
Conduct problems	4.80 (4.82)	4.20 (4.43)	10.19 (5.77)	9.00 (5.44)
Less severe delinquency	0.98 (2.59)	0.38 (1.26)	8.77 (9.65)	3.65 (6.26)
Severe delinquency	2.59 (3.85)	0.72 (1.85)	9.17 (7.88)	4.46 (5.16)
Affiliation with delinquent peers	18.71 (6.06)	17.28 (5.66)	25.11 (7.07)	25.21 (6.84)

*M* mean, *SD* standard deviation

Tables 3 and 4 present effect sizes for each dependent variable, as well as the summary statistics. With regard to internalizing problems (Table 3), the main effect for inhalant use (*η*^2^ = 0.026, *p* < 0.001) was significant, suggesting increasing levels of internalizing psychopathology in inhalant users. The main effect for gender (*η*^2^ = 0.002, *p* > 0.05) was not significant, which indicates that there were no gender differences in relation to internalizing problems. The interaction effect for inhalant use × gender was not significant for any of the internalizing variables, except for posttraumatic stress (*η*^2^ = 0.002, *p* < 0.05), which means that the overall general pattern of internalizing problems in relation to inhalant use did not differ between boys and girls. Finally, the main effect for age was significant (*η*^2^ = 0.024, *p* < 0.001), suggesting that the levels of internalizing problems increased with age. The post hoc univariate effects for internalizing problems in relation to inhalant use were significant for depressive symptoms (*η*^2^ = 0.008, *p* < 0.001), and posttraumatic stress (*η*^2^ = 0.011, *p* < 0.001), but not for anxiety (*η*^2^ = 0.000, *p* > 0.05). The post hoc univariate effects in relation to inhalant use were also significant for academic motivation (*η*^2^ = 0.007, *p* < 0.001) and SDQ impairment (*η*^2^ = 0.016, *p* < 0.001) which indicates poorer school achievement and increasing difficulties with emotions, concentration and a higher degree of distress in inhalant users.

**Table 3:** Effect sizes for internalizing dependent variables (*η*^2^, p) and summary statistics (Wilks’ lambda, F(df), *η*^2^, p)

	Depression	Anxiety	Posttraumatic stress	Academic motivation	SDQ impairment	Summary statistics
Age	0.013, < 0.001	0.001, ns	0.001, ns	0.006, < 0.001	0.009, < 0.001	0.976, 13.02 (5, 2595), 0.024, < 0.001
Inhalant use	0.008, < 0.001	0.000, ns	0.011, < 0.001	0.007, < 0.001	0.016, < 0.001	0.974, 13.78 (5, 2595), 0.026, < 0.001
Gender	0.002, < 0.05	0.001, ns	0.001, ns	0.000, ns	0.000, ns	0.998, 1.24 (5, 2595), 0.002, ns
Inhalant use by gender	0.001, ns	0.001, ns	0.002, < 0.05	0.000, ns	0.000, ns	0.997, 1.66 (5, 2595), 0.003, ns

**Table 4:** Effect sizes for externalizing dependent variables (*η*^2^, *p*) and summary statistics (Wilks’ lambda, *F* (*df*), *η*^2^, *p*)

	Alcohol use	Alcohol-related problems	Marijuana use	Substance use-related problems	Conduct problems	Less severe delinquency	Severe delinquency	Affiliation with delinquent peers	Summary statistics
Age	0.091, < 0.001	0.015, < 0.001	0.020, < 0.001	0.007, < 0.001	0.036, < 0.001	0.005, < 0.001	0.003, < 0.01	0.056, < 0.001	0.883, 42.64 (8, 2582), 0.117, < 0.001
Inhalant use	0.020, < 0.001	0.062, < 0.001	0.088, < 0.001	0.221, < 0.001	0.039, < 0.001	0.154, < 0.001	0.091, < 0.001	0.049, < 0.001	0.747, 109.08 (8, 2582), 0.253, < 0.001
Gender	0.000, ns	0.010, < 0.001	0.015, < 0.001	0.017, < 0.001	0.002, < 0.05	0.048, < 0.001	0.040, < 0.001	0.001, ns	939, 21.06 (8, 2582), 0.061, < 0.001
Inhalant use by gender	0.000, ns	0.004, < 0.001	0.008, < 0.001	0.013, < 0.001	0.000, ns	0.030, < 0.001	0.008 < 0.001	0.001, ns	0.962, 12.92 (8, 2582), 0.038, < 0.001

For externalizing problems (Table 4), the main effect for inhalant use was significant (*η*^2^ = 0.253, *p* < 0.001), suggesting higher levels of externalizing problems in inhalant users (see Table 2 for means and SDs). However, the associations were much stronger, with externalizing problems explaining 25% of the variance in inhalant use (as opposed to the 3%, explained by internalizing problems). Here, we also observed a significant main effect for gender (*η*^2^ = 0.061, *p* < 0.001), as well as a significant interaction effect for inhalant use × gender (*η*^2^ = 0.038, *p* < 0.001), explaining 6 and 4% of the variance in externalizing problems, respectively. The post hoc univariate effects for inhalant use in relation to externalizing problems were significant for all studied determinants, such as alcohol (*η*^2^ = 0.020, *p* < 0.001) and marijuana use (*η*^2^ = 0.088, *p* < 0.001), conduct problems (*η*^2^ = 0.039, *p* < 0.001), less severe (*η*^2^ = 0.154, *p* < 0.001) and severe delinquency (*η*^2^ = 0.091, *p* < 0.001). In addition, the post hoc univariate effect for alcohol-related problems (*η*^2^ = 0.062, *p* < 0.001) and substance use-related problems (*η*^2^ = 0.221, *p* < 0.001) in relation to inhalant use was also significant indicating higher problem levels with increased use of inhalants.

As the differences by inhalant use and gender could have been masked using a MANCOVA analysis (i.e. by simultaneously assessing all three outcomes in one model), we also attempted to examine each outcome separately to determine whether the patterns that were obtained from the MANCOVA analysis were also obtained for each outcome individually. The results from the Uni ANCOVA were largely the same as those from the MANCOVA analysis (data not shown).

## Discussion

The prevalence of inhalant use among adolescents in our study ranged from 3.4% among girls to 6.1% among boys. These figures are somewhat lower than those seen earlier in both English [20] and Russian language reports (e.g. data from the Ministry of Education, 2003). Although evidence suggests that the prevalence rates of inhalant use in specific adolescent populations in Russia, such as street children [35], or juvenile delinquents [36], may be substantially higher, the discrepancy in rates between studies highlights the need for further research in this area.

The association observed between inhalant use and negative health and psychosocial effects is consistent with the results from studies in other countries [9–12, 14, 37]. As suggested earlier, inhalant users often experience academic problems which can include a scholastic decline [38] and which can sometimes lead to them dropping out of school altogether [39, 40]. In our study, inhalant users had lower academic motivation which indirectly supports the previously reported association between substance use and adolescents’ poorer school achievements. Several studies have pointed to the possible bidirectionality of the association between low academic motivation and substance use [41]. Adolescents may use inhalants to cope with their concerns over academic failure and as a result lose their academic motivation [42, 43]. Alternatively, substance use may be a predisposing factor for difficulties at school, through reduced academic motivation [44] or because of substantial cognitive problems developing as a result of inhalant use [11].

Consistent with earlier research [3], in the present study, inhalant use was also associated with depression and stress. It is possible that adolescents might use substances in an attempt to overcome negative moods and decrease perceived stress. The potential complexity and circularity of these relationships requires further research to elucidate these associations so that evidence-based interventions can be formulated to prevent adolescent depression and stress, and decrease risky behaviors.

While it is difficult to come to any direct conclusions about the clinical mental health needs of adolescents using inhalants in the present study, our findings nevertheless suggest that the problem levels of these youth are significantly higher than those in the general population. The greater mental health needs of inhalant users in general are well documented, with some research even suggesting that internalizing psychopathology tends to increase along with the increasing frequency of inhalant use in this group [7]. Similar to those individuals with a clinical diagnosis of inhalant abuse which is characterized by a substantial degree of functional impairment and of extensive comorbidity [45], the inhalant users in this study indicated a greater level of perceived functional impairment, associated with comorbid mental health problems.

Inhalant users in our study demonstrated a worrying profile in terms of having a wide range of behavioral problems. Inhalant use was associated with more externalizing problems, and this association was much stronger compared to that with internalizing problems. Inhalants users were significantly more likely to engage in delinquent behaviors, use other types of illegal substances and experience greater problems associated with substance use, as well as associate with delinquent peers. It is well documented that the above problems commonly co-occur [46, 47]. Inhalant users with behavioral problems may be especially vulnerable to the development of other types of psychopathology and of substance abuse [48, 49] with a high degree of comorbidity between behavioural problems and substance use disorders having been reported in several studies [46, 47]. This highlights the complexity of planning treatment efforts for inhalant users and the need to address a broad range of problems. In addition, it should also be remembered that not only engaging in juvenile delinquency strongly predisposes adolescents to use substances [50], including inhalants, but that chronic inhalant use creates an additional burden in terms of long-lasting sequela, such as increased cognitive impairment, problems with impulse control, and mental health problems [11, 12], which in turn increase the risk for repeated antisocial involvement.

The association observed between inhalant use and marijuana use and substance use-related problems is consistent with the findings from earlier studies [51]. As suggested previously [52, 53], compared with adolescents who use inhalants but who do not use marijuana, adolescents who use both substances have a particularly high rate of substance use disorders, including alcohol use disorders, and thus, represent a severe set of drug users, who require increased attention and multifaceted intervention efforts from health care providers and the school system alike.

The interaction effect for inhalant use by gender was not significant for anxiety and depression in our study suggesting a similar pattern of internalizing problems in relation to inhalant use among boys and girls. However, a gender-specific difference with regard to posttraumatic stress was observed, where stress levels were lower in non-using boys than in non-using girls, whereas in users, the opposite result was observed. Although greater traumatization in inhalant users has been well documented in previous research [36], to our knowledge, higher posttraumatic stress in male users has not been reported previously. This finding can, however, be tentatively explained by the often much heavier use of inhalants in boys [54, 55], as those under the influence of substances are generally at greater risk for being exposed to trauma. The gender differences relating to inhalant use were most pronounced for externalizing problems. This finding may relate to the substantially higher prevalence rates of externalizing behaviors in male as compared to female adolescents [56].

As demonstrated in earlier studies, inhalant use has a substantial effect on the presence of psychopathology in youth. Since many solvent abusers begin experimentation in the pre-teenage years, preventive educational programs are best begun in the primary school grades. Continuing efforts are needed to educate adolescents, parents, teachers, physicians, service providers, and policy makers about the dangers and health risks of inhalant use [57]. Almost unequivocally, prevention is considered the most effective strategy and should be comprehensive, evidence- and community-based, involving not only users and family members, but also peers and schools. Our results highlight the need to identify inhalant use and its comorbid disorders as early as possible to facilitate timely interventions to enhance psychosocial health. Since volatile substances are legally available, cheap, easy to find, and are highly addictive, inhalant use will continue until appropriate methods of prevention and intervention measures are devised. Inhalant use offers a distinct challenge to the health care provider, who needs to be made more aware of the prevalence of inhalant use in the populations they serve, commonly abused products, and the medical consequences of intoxication and habitual use.

## Limitations

This study has several limitations that should be mentioned. First, our findings are based exclusively on adolescent self-reports, which are subject to recall and reporting biases. In addition, some information, such as the relatively low reported prevalence of inhalant use might have been due to underreporting because of the teachers’ presence in the classroom while the students completed the questionnaires. Second, as this study was cross-sectional causal links could not be established between any of the variables. Third, we also lacked information about the age of onset of inhalant use and on factors associated with the initiation of inhalant use, which may have further elucidated the relation between inhalant use and negative psychosocial outcomes. Fourth, the small number of inhalant users meant that it was not possible to examine the difference between frequent and occasional use. Finally, we had no information on other factors which might have been important for our results such as parental mental health and their substance/inhalant use.
